# SM22α suppresses cytokine-induced inflammation and the transcription of NF-κB inducing kinase (*Nik*) by modulating SRF transcriptional activity in vascular smooth muscle cells

**DOI:** 10.1371/journal.pone.0190191

**Published:** 2017-12-28

**Authors:** Xiaohua Dai, Devi Thiagarajan, Jingye Fang, Jianbin Shen, Neeraja Priyanka Annam, Zhao Yang, Hong Jiang, Donghong Ju, Youming Xie, Kezhong Zhang, Yan Yuan Tseng, Zhe Yang, Arun K. Rishi, Hui J. Li, Maozhou Yang, Li Li

**Affiliations:** 1 Department of Internal Medicine, Wayne State University, Detroit, Michigan, United States of America; 2 Center for Molecular Medicine and Genetics, Wayne State University, Detroit, Michigan, United States of America; 3 Department of Biochemistry and Molecular Biology, Wayne State University, Detroit, Michigan, United States of America; 4 Department of Oncology, Barbara Ann Karmanos Cancer Institute, Wayne State University, Detroit, Michigan, United States of America; 5 Cardiovascular Research Institute, Wayne State University, Detroit, Michigan, United States of America; 6 John D. Dingell VA Medical Center, Detroit, Michigan, United States of America; 7 Department of Medicine, University of Massachusetts, Worcester, Massachusetts, United States of America; 8 Bone and Joint Center, Henry Ford Hospital, Detroit, Michigan, United States of America; University of Nevada School of Medicine, UNITED STATES

## Abstract

Vascular smooth muscle cell (VSMC) phenotypic modulation is characterized by the downregulation of SMC actin cytoskeleton proteins. Our published study shows that depletion of SM22α (aka SM22, Transgelin, an actin cytoskeleton binding protein) promotes inflammation in SMCs by activating NF-κB signal pathways both in cultured VSMCs and in response to vascular injury. The goal of this study is to investigate the underlying molecular mechanisms whereby SM22 suppresses NF-κB signaling pathways under inflammatory condition. NF-κB inducing kinase (*Nik*, aka *MAP3K14*, activated by the LTβR) is a key upstream regulator of NF-κB signal pathways. Here, we show that SM22 overexpression suppresses the expression of NIK and its downstream NF-κB canonical and noncanonical signal pathways in a VSMC line treated with a LTβR agonist. SM22 regulates NIK expression at both transcriptional and the proteasome–mediated post-translational levels in VSMCs depending on the culture condition. By qPCR, chromatin immunoprecipitation and luciferase assays, we found that *Nik* is a transcription target of serum response factor (SRF). Although SM22 is known to be expressed in the cytoplasm, we found that SM22 is also expressed in the nucleus where SM22 interacts with SRF to inhibit the transcription of *Nik* and prototypical SRF regulated genes including *c-fos and Egr3*. Moreover, carotid injury increases NIK expression in *Sm22*^-/-^ mice, which is partially relieved by adenovirally transduced SM22. These findings reveal for the first time that SM22 is expressed in the nucleus in addition to the cytoplasm of VSMCs to regulate the transcription of *Nik* and its downstream proinflammatory NF-kB signal pathways as a modulator of SRF during vascular inflammation.

## Introduction

Vascular smooth muscle cell (VSMC) phenotypic modulation plays critical roles in the pathogenesis of vascular diseases such as atherosclerosis and aneurysms [1, 2]. SMC phenotypic modulation is accompanied by the down regulation of actin cytoskeleton proteins including smooth muscle α-actin (SMA), SM22α, Calponin and smooth muscle myosin heavy chain (SMMHC). Extensive studies have characterized the central role of SRF in SMC phenotypic switching from the differentiated state to a variety of dedifferentiated states involved in VSMC proliferation, migration, inflammation and calcification [1–3]. However, the underlying molecular mechanisms that regulate these pathophysiological processes remain largely unknown.

SM22 (aka Transgelin), a transformation and shape-change sensitive actin cross-linking/gelling protein [4], is known to be a VSMC differentiation marker during embryogenesis as well as in the adult [5]. SM22 is a highly conserved protein ranging from Yeast, *C*.*elegans* to humans [4]. SM22 contains several distinct domains including a C-terminal actin binding domain (ABD) and the Calponin homology domain (CH) [6, 7]. In addition to actin binding, the ABD of SM22 facilitates the bundling of F-actin and the formation of cytoskeletal structures such as stress fibers [6].

The viability and fertility of *Sm22*^-/-^ mice indicate that the function of SM22 can be compensated for during vascular development [8–10]. However, under stress conditions, loss of SM22 leads to enlarged atherosclerotic lesions in high fat diet treated ApoE^-/-^ mice[11], and after arterial injury there is increased inflammation, oxidative stress, proliferation, and medial chondrogenesis [12–16]. These results suggest that SM22 has a protective role in VSMC phenotypic modulation. Indeed, SM22 dysregulation has been reported in various human diseases such as atherosclerosis, aneurysms, autoimmune diseases and various cancers [17–21]. Inflammation is the key trigger in many of these pathological conditions. NF-κB-mediated signal pathways play important roles in inflammation.

NF-κB proteins comprise a family of structurally related transcription factors regulating immune and inflammatory responses to control growth, development and apoptosis [22, 23]. Under normal conditions, NF-κB proteins are retained in the cytoplasm. Under inflammation conditions, proinflammatory cytokines or stress signals induce IKK-mediated canonical and NIK-mediated non-canonical signal pathways, resulting in the degradation of IKB, proteolysis of p105 and p100 into p50 and p52 respectively, NF-κB nuclear translocation and transcriptional activation of proinflammatory genes [23, 24]. These transcription factors remain persistently active in diseased states including cancer, arthritis, inflammation and cardiovascular diseases. The crosstalk between canonical and noncanonical signal pathways is prevalent under inflammatory conditions and the composition of NF-κB complexes in the nucleus is cell-context dependent.

Our published study shows that SM22 deficiency promotes inflammation in VSMCs by activating both the IKB degradation mediated canonical and p100 degradation-mediated noncanonical NF-κB signal pathways *in vitro*, and the noncanonical pathway is significantly activated two weeks after carotid injury [12]. This result prompted us to investigate the molecular mechanisms whereby SM22 suppresses NF-κB mediated inflammation. A subsequent independent study shows that SM22 inhibits TNF-induced inflammation via inhibiting IKB degradation [13]. Here, we report a new mechanism whereby SM22 suppresses the expression of NIK (NF-κB induced kinase, aka MAP3K14), an upstream regulator of the NF-κB signaling pathways. In elucidating the underlying molecular mechanism, we unexpectedly discovered that SM22 is present in the nucleus in addition to the cytoplasm. Moreover, we found that SM22 acts as a SRF modulator to regulate the transcriptional activities of SRF on the transcription of genes involved in inflammation.

## Materials and methods

### Tissue culture

The VSMC cell line PAC1 (pulmonary artery derived smooth muscle cells [25, 26]) was maintained in Dulbecco’s modified Eagle medium (DMEM) supplemented with 10% fetal bovine serum (FBS), penicillin (100 U/mL) and streptomycin (100 g/mL) at 37°C in a humidified 5% CO_2_ as described before.

Subconfluent PAC1 cells in 10% FBS were transiently transfected with indicated plasmids using Lipofectamine and/or Plus transfection reagents (Invitrogen) for 24 hours (hr) following the manufacturer’s instruction. A recombinant human LTβR agonistic antibody (lymphotoxin beta receptor Fc chimera, referred as LTβR-Fc, R& D Systems, Cat #629-LR-100) was reconstituted in sterile PBS. The LTβR agonist LTβR-Fc at 10ng/ml was used to induce the activation of LTBR signal pathway by adding to PAC1 cells in 10% FBS [27]. In all figures throughout this manuscript, LTβR-Fc refers to this LTβR agonist. For inhibitor studies, transfected PAC1 cells were treated with proteasome inhibitor MG132 (1μM, Sigma) for 24 hrs or Actinomycin D (5μg/ml, Sigma) for 4 hrs after transient transfection.

### Luciferase reporter assay

PAC1 cells were plated onto 96 well plates one day before transient transfection. Cignal NF-κB reporter assay luciferase kit (SABiosciences, Cat # CCS-013L) was used per manufacturer’s protocol. Subconfluent PAC1 cells were transfected with 50ng of corresponding plasmids along with 1ng of pRL-CMV plasmid encoding Renilla luciferase (internal control) using Lipofectamine (Invitrogen). 24hrs post transfection, cells were treated with LTβR-Fc for 24hrs. Luciferase activities were measured using the dual luciferase assay system as described before [16] and readings were measured using Veritas microplate luminometer. Relative luciferase activity was normalized to Renilla luciferase activities (the internal control). Experiments were performed at least three times independently and the results are presented with standard errors.

### Plasmids

pCMV6-XL5-hSM22 was purchased from Origene Inc (#sc118118). To construct the C-terminal V5 tagged human *SM22* and its truncation mutants, an EcoRI-XhoI-BamHI-KpnI-V5-XbaI fragment was subcloned into EcoRI and XbaI sites of pcDNA3.1. The resulting construct was cleaved with HindIII and XhoI followed by insertion of the PCR fragments containing the full length V5-tagged human *SM22* or its truncation mutants. Clones were verified by sequencing (Genewiz Technologies, INC).

The pCGN-SRF plasmid and the luciferase reporter driven by the 4xCArG boxes from the *fos* promoter were generous gift from Dr. Eric Olson [28, 29]. The 1.4kb mouse *Egr3* promoter (upstream of the ATG) containing the CArG box (CCATATATGG) was PCR cloned into the XhoI/HindIII sites of pGL3-basic luciferase vector (Promega). About 2kb rat *Nik* promoter (upstream of the ATG) containing the CArG box (CCAACAATGG) was PCR cloned into the NheI/HIII sites of pGL3-basic luciferase vector (Promega). The CArG box mutant (TTAACAATT) was subsequently generated as the *Nik*2kbCArGmut-luc. All constructed luciferase reporters containing the promoter fragment or the CArG box mutant were verified by sequencing (Genewiz Technologies, INC). All plasmids DNA were prepared for transfection using the plasmid Prep kit (Qiagen).

### Quantitative real time RT-PCR (qPCR)

Total RNA from PAC1 cells was extracted using RNeasy Kit (Qiagen) according to the manufacturer’s protocol. cDNA was synthesized from 1ug of total RNA using the Superscript II reverse transcriptase kit (Invitrogen). qPCR was performed with StepOne Plus system (Applied Biosystems) using Fast Sybr green master mix (Applied Biosystems). Relative mRNA level was normalized using Gapdh and SnRNA U6 as internal controls. The sequences of the forward and reverse primers used in qPCR assays are listed in the Table (S2 Table).

### Western blot (WB) assay

Total cell protein lysate were made using M-PER Mammalian Protein extraction reagent (Thermo Scientific) according to the manufacturer’s protocol. For nuclear and cytoplasmic fractions, NE-PER Nuclear and Cytoplasmic Extraction Reagents (Thermo Scientific) were used per manufacturer’s protocol. Proteins were quantified with Quant IT protein assay kit (Invitrogen) and measured using Qubit flurometer (Invitrogen). Equal amounts of proteins were subjected to electrophoresis on 4–12% Bis-Tris NuPAGE Mini gel (Invitrogen) followed by transfer to PVDF membrane (Millipore). The membrane was blocked with 5% non-fat milk for 90 min and incubated with primary antibody overnight at 4°C. After incubation with HRP conjugated secondary antibody for 1hr, the membrane was subjected to chemiluminescence detection using Super signal West Pico Chemiluminescent Substrate (Thermo Scientific). The blots were exposed to HyBlot CL film (Denville) for signal visualization.

To ensure equal loading, the membrane was probed with GAPDH antibody. Antibodies used for detection were SM22 (Abcam, ab14106, 1:2500), GAPDH (Santa Cruz, sc-25778, 1:1000), SDF-1 (Santa Cruz, sc6193, 1:2000), ICAM-1 (Santa Cruz, sc1511, 1:1000), p105/p50 (Abcam, ab32360, 1:2000), IκBα (Santa Cruz, sc-371, 1:1500), p100/p52 (Abcam, ab31409, 1:2000, not available now), NF-κB inducing kinase (Santa Cruz, sc-7211, 1:1000), Lamin B (Santa Cruz, sc-6217, 1:1500), p65 (Santa Cruz, sc109, 1:1000), smooth muscle-α actin (Abcam, ab5694, 1:3000), SRF (Santa Cruz, sc-335, 1:2000). The corresponding secondary antibodies used were HRP conjugated anti-rabbit IgG, anti-mouse IgG (Santa Cruz), or anti-goat IgG (Thermo Scientific). Protein expression levels are quantified by normalizing to GAPDH using the Image J software (NIH).

### Immunofluorescence

For immunostaining, cells were fixed with 4% paraformaldehyde at room temperature for 10 min. After washing cells briefly three times with PBS for 5 minutes, cells were blocked and permeabilized with 0.2% Tritox-100 and 10% chicken serum in PBS for 1 hour at 37°C then followed by primary antibody incubation for 1 hour at 37°C. Cells were washed three times with 1X PBS, then conjugated with Alexa fluor 488 (Invitrogen Molecular Probes) and incubated for 1 hour at 37°C. After washing 3 times with PBS for 5 minutes, slides were mounted with antifade mounting medium with DAPI (Vectorlabs) and examined under a Leica DM4000B microscope. Primary antibody was against SM22 (Abcam, ab14106, 1:200).

### siRNA transfections

For siRNA experiments, *Sm22* siRNA oligos (GCAGAUCAUCAGUUAGAAAGGGAAG, MMC.RNAI.N011526.5.1) and the scramble siRNA control was obtained from IDT and suspended in RNAse free water and transfected using DharmaFECT (Thermoscientific) per manufacturer’s protocol. 48 hours after transfection, total RNAs were isolated for real-time PCR assay and proteins were extracted for Western blotting.

### Co-immunoprecipitation (Co-IP) assay

Protein lysates were made from PAC1 cells (the whole cell, cytoplasm and nuclei) using M-PER Mammalian Protein Extraction Reagent and NE-PER Nuclear and Cytoplasmic Extraction Reagents (Thermo Scientific). 400μg of protein was used for the precipitation. 2μg of IgG was used for pre-clearing. For immunoprecipitation, 4 μg of antibody was added followed by overnight incubation at 4°C with constant rotation. The pull down fraction was washed three times using the same lysis buffer and subjected to western blot. The precipitated proteins were detected using the clean Blot^™^ IP detection Kit (Thermo Scientific).

### ChIP assay

ChIP assays were performed using the Pierce^™^ Agarose ChIP Assay Kit (Thermo Scientific, Catelog # PI26156) according to manufacture instruction. The cross-linked chromatin from subconfluent PAC1 cells in 10% FBS was immunoprecipitated using anti-SRF antibodies (Santa Cruz, sc-335), followed by qPCR using primers specific to the *Nik* promoter in a region containing the CArG box (Forward primer: 5′—TGTTCAGCCCATTTTTAGGC -3′, Reverse primer: 5′- TTTAGCATTGTGCGAGTGTC -3′) (S2 Table).

### Mouse carotid artery injury model and adenoviral gene transfer

Both mouse carotid denudation and ligation were used as carotid injury models. The protocols were approved by the Animal Investigation Committee at Wayne State University. The animal procedures performed conform with the NIH guidelines (Guide for the care and use of laboratory animals). Generation and characterization of *Sm22*^−/−^ mice at mixed C57BL/6 x SV129 genetic background were described previously [10, 12]. The mouse carotid denudation injury model was described before [12, 14].

For the mouse carotid ligation injury model, *Sm22*^−/−^ mice were obtained by breeding *Sm22*^+/−^ mice. All mice were healthy with no bacterial/viral infection as regularly monitored by the Animal Investigation Committee at Wayne State University. Male *Sm22*^−/−^ mice at 18–20 weeks of age were used to be anaesthetized by 2% Tribromoethanol (Avertin, 250 mg/kg body weight) intraperitoneally. The carotid ligation procedure was performed as described before [30]. Briefly, the left common carotid of mice under anesthesia was dissected and ligated near the carotid bifurcation into internal and external carotid artery branches. The right common carotid served as the uninjured control. To minimize the pain caused by the procedure, the non-steroidal anti-inflammatory agent, Carprofen (at 5 mg/kg in 0.5 ml of normal saline) was then administered intraperitoneally. All mice were closely monitored during the entire process until they regained consciousness.

For adenoviral transduction in ligated carotids, human SM22 cDNA in pCMV-IRES-eGFP adenovirus (Ad-SM22-GFP) or its control (Ad-GFP) (Cyagen Biosciences Inc.) in pluronic F127 gel (Sigma, P2443, BASF, 25% w/v in sterile saline) (1×10^10^ pfu/ml) were prepared and maintained at 4°C as described before [31]. To avoid the hormonal effect of female on the injury responses, only male mice were used in the study. 14 male *Sm22*^−/−^ mice at 18–20 weeks of age were randomly divided into two groups (n = 7 for each group) for carotid ligation as described above. Upon carotid ligation in the laboratory, 200ul of adenovirus pluronic gel was applied to the perivascular surface of the carotid artery. Following the rapid solidification of the gel around the artery at body temperature, the wound was closed and animals were allowed to recover before sending back to animal room for housing.

Two weeks after the surgery, mice were anesthetized intraperitoneally using 2% Tribromoethanol (Avertin, 250 mg/kg body weight) followed by cardiac punctuation for terminal single blood collection. After death, the carotids from proximal to ligature to the aortic arch were harvested for embedding in OCT medium (Tissue-Tek), with triplicate sections on each slide at 6 μm thickness. Morphometric analyses following H&E staining were performed to scan the vessel wall remodeling. Sections at about the same location away from the bifurcation of left and right common carotid arteries were used for gene expression analyses by assessing the staining intensity detected by indicated antibodies in the IHC assay.

### Immunohistochemistry (IHC) assay

IHC was performed on the OCT sections using VECTASTAIN Elite ABC Kit (Vectorlabs). Briefly, air-dried slides were fixed in methanol containing 0.3% H_2_O_2_ for 10 min at -20°C, then serum-blocked for 20 min at room temperature, followed by antibodies incubation overnight at 4°C. Slides were washed three times with 1X PBS, then conjugated with secondary antibody and incubated for 1 hour at 37°C. After washing 3 times with PBS for 5 minutes, the tissues were stained with ABC reagent and DAB substrate according to manufacturer’s protocol. Slides were counterstained with hematoxylin. The antibodies were against NIK (Santa Cruz, sc-7211, 1:100), and VCAM-1 (Santa Cruz, sc1504, 1:100). We assessed the staining intensity with blinded genotype and treatment. Quantification was performed using the integrative optical density function in Image-Pro software.

### Statistical analysis

The values presented are means ± standard errors of the means (SEMs). Differences between two different groups were evaluated by unpaired Student *t* test using Prism software (GraphPad). Differences with p values < 0.05 were considered statistically significant.

## Results

### Overexpression of SM22 suppresses the expression of proinflammatory genes in VSMCs under inflammatory condition

**Fig 1:**
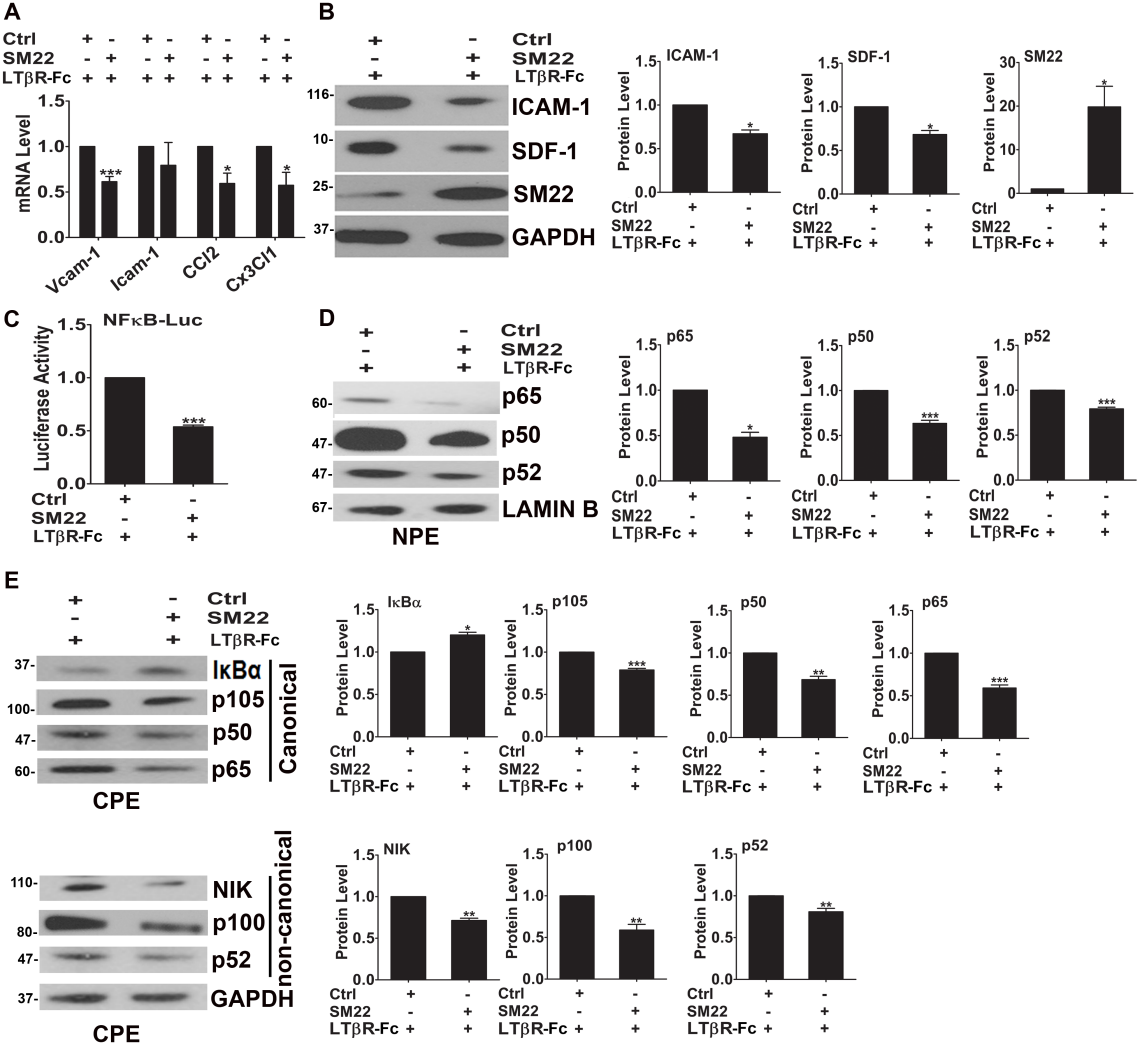
SM22 suppresses NF-κB signal pathways in VSMCs under inflammation condition. PAC1 SMC cells were transfected with either SM22 expression plasmid or its empty vector control (Ctrl) plasmid followed by the treatment of LTβR-Fc (a LTβR agonistic antibody that activates LTβR-mediated NF-κB signal pathways) for 24 hours. (A) SM22 overexpression suppressed the transcription of proinflammatory markers by qPCR assays. n = 4. (B) SM22 overexpression suppressed the expression of proinflammatory proteins ICAM-1 and SDF-1 by WB assays. Quantification of protein levels after normalizing to GAPDH is shown on the right. n = 3. (C) SM22 overexpression suppressed luciferase activities of NF-κB site-luc reporter by luciferase assays. n = 3. (D) SM22 overexpression suppressed the expression of NF-κB protein p65, p50 and p52 in the nucleus by WB assays using the nuclear protein extract (NPE). Quantification of protein levels after normalizing to LAMIN B (a nuclear protein marker) is shown on the right. n = 3. (E) SM22 overexpression modulated the expression of key components of NF-κB canonical and non-canonical signal pathways in the cytoplasm by WB assays using the cytoplasmic protein extract (CPE). Quantification of protein levels after normalizing to GAPDH in WB is shown on the right of the representative WB images. n = 3. The molecular weight markers are indicated on the left side of the images. Note: *, **, and *** indicate *p*<0.05, *p*<0.01, and *p*<0.001 respectively vs. the control (Ctrl).

Our published study shows that the NF-κB noncanonical pathway is significantly activated in SM22 knockout mice two weeks after carotid injury [12]. To determine the underlying molecular mechanism whereby SM22 inhibits the NF-κB noncanonical pathway under inflammatory condition, we used a classical lymphotoxin beta (LTβ) proinflammatory cytokine to induce the noncanonical NF-κB pathway in SMCs. In this report, we used a LTβ receptor agonistic antibody (indicated as LTβR-Fc) [27] to stimulate LTβR activated NF-κB signal pathways in VSMCs. In LTβR-Fc treated PAC1 cells (a VSMC cell line), we found that exogenous overexpression of SM22 reduced LTβR activation-induced transcription of proinflammatory genes *Vcam1*, *Icam1*, *Ccl2* and *Cx3cl1* by the qPCR assay (Fig 1A) and the expression of ICAM1 (a representative canonical NF-κB target protein) and SDF-1 (a representative non-canonical NF-κB target protein) by western blotting (WB) assay (Fig 1B). However, in the absence of LTβR-Fc, SM22 overexpression did not suppress inflammation in PAC1 cells (S1 Fig): this is consistent with a published data showing that SM22 overexpression did not suppress inflammation in primary VSMCs even though it suppressed TNFα-induced inflammation [13].

To determine the effect of SM22 on the transcriptional activities of NF-κB, the key regulator of inflammation gene transcription under inflammatory condition, we performed luciferase assays in LTβR-Fc treated PAC1 cells and show that SM22 overexpression suppressed the transcriptional activities of a luciferase reporter driven by a tandem linked NF-κB bind sites (Fig 1C).

Our previous studies show that knockdown SM22 induces the decrease of IKB and p100 in the cytoplasm and the increase of p65 (RELA) and p52 (NFKB2) in the nucleus in VSMCs [12]. Consistent with this study using the SM22 knockdown approach, we found that SM22 overexpression suppressed LTβR-induced NF-κB proteins involved in both canonical (p50, p105, and p65) and noncanonical (NIK, p100 and p52) pathways by WB using nuclear protein extracts (NPE, Fig 1D) and cytoplasmic protein extracts (CPE, Fig 1E) from LTβR-Fc treated PAC1 cells. Please note that SM22 overexpression increased the expression of IκBα in the cytoplasm (Fig 1E). This result is consistent with current studies showing that activation of LTβR induces both canonical and noncanonical NF-κB pathways [32, 33]. Since SM22 overexpression suppressed both canonical and noncanonical NF-κB signal pathways under inflammatory condition, we speculated that SM22 may act upstream in the activation of NF-κB pathways.

### SM22 suppresses the expression of *Nik*, an upstream regulator of the NF-κB signal pathways

**Fig 2:**
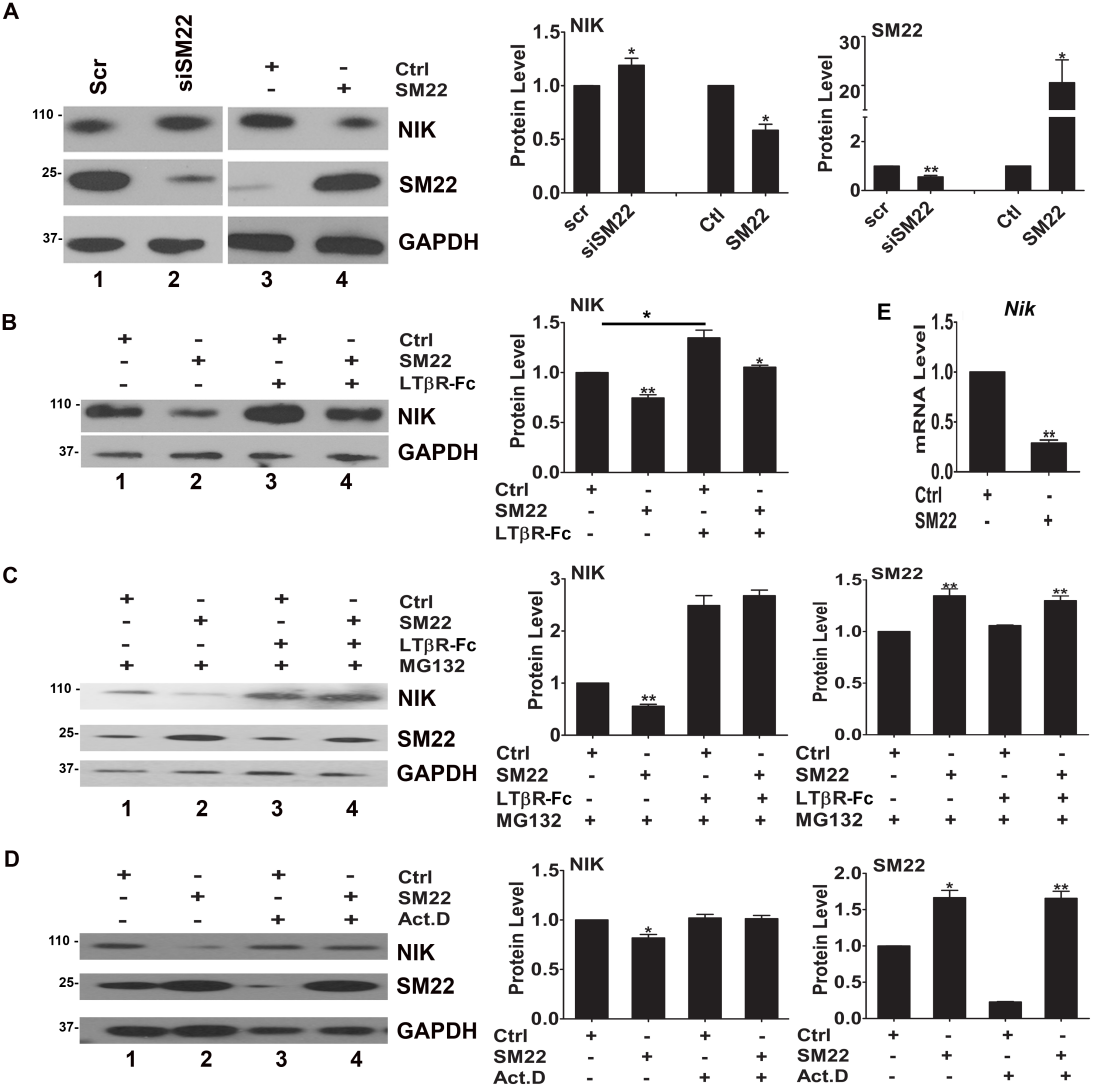
SM22 regulates NIK expression at both proteasome-mediated posttranslational and transcriptional levels in VSMCs depending on the culture condition. PAC1 cells were treated with or without LTβR-Fc for 24 hours as indicated. (A) Knockdown SM22 increased NIK expression while SM22 overexpression reduced NIK expression by WB analysis. Quantification of protein levels after normalizing to GAPDH is shown on the right. (B) LTβR-Fc treatment for 24 hours induced NIK expression and SM22 overexpression inhibited both basal and LTβR-induced NIK expression. (C) In the presence of proteasome inhibitor MG132, SM22 inhibited the expression of NIK, but such inhibition was not detected when PAC1 cells were treated with LTβR-Fc for 24 hours. (D) In the presence of transcription inhibitor Actinomycin D for 4 hours, SM22 overexpression failed to inhibit the expression of NIK. (E) SM22 overexpression inhibited the transcription of *Nik* by qPCR assay. n = 3. Note: Quantification of protein levels after normalizing to GAPDH in WBs is shown on the right of the representative WB images. n = 3. The molecular weight markers are indicated on the left side of the WB images. *, **, and *** indicate *p*<0.05, *p*<0.01, and *p*<0.001 respectively vs. the control (Ctrl).

One possible candidate acting upstream of NF-κB signal pathways is NF-κB inducing kinase (NIK, aka MAP3K14), that can be activated by the lymphotoxin beta receptor (LTβR) to stimulate the noncanonical NF-κB signal pathway as well as the canonical pathway [33]. Indeed, we found that SM22 overexpression decreased NIK expression while SM22 depletion by *siSm22RNA* increased NIK expression in PAC1 cells (Fig 2A). This result indicates that SM22 overexpression suppresses the expression of NIK (an upstream activator of NF-κB pathways) to downregulate both the canonical and non-canonical NF-kB signal pathways as well as their downstream targets.

As expected, LTβR activation increased NIK expression in PAC1 cells treated with LTβR-Fc by WB (Fig 2B lane 1&3). We also found that SM22 inhibited NIK expression under both basal and LTβR-Fc treated conditions (Fig 2B lane 2&4 comparing to lane 1&3). This prompted us to explore the underlying molecular mechanisms whereby SM22 inhibits NIK expression.

### SM22 regulates NIK expression through different mechanisms under basal and inflammatory conditions

LTβR activation is known to increase NIK expression by repressing proteasome-mediated NIK degradation [32]. To determine whether SM22 inhibits NIK expression by increasing proteasome degradation [34], we examined the expression of NIK in PAC1 cells treated with proteasome inhibitor, MG132. WB assays showed that SM22 overexpression inhibited NIK expression in spite of the presence of MG132 (Fig 2C, Lane 1&2): this result suggests that SM22 may inhibit NIK expression through a mechanism independent of the ubiquitin-mediated proteasome degradation under basal condition. However, blocking protein degradation by MG132 relieved the inhibitory effect of SM22 on LTβR-Fc-induced NIK expression by WB (Fig 2C Lane 3&4): this result suggests that the inhibitory effect of SM22 on LTβR-Fc-induced NIK expression may involve in protein degradation under the inflammatory condition. Therefore, SM22 inhibits NIK expression through different mechanisms under basal and inflammatory conditions.

### SM22 regulation of NIK is transcriptional

SM22-regulated NIK expression could occur at either post-translational and/or transcriptional levels. Since SM22 overexpression does not affect NIK degradation under basal condition (see above Fig 2C), we thus tested whether SM22 reduces NIK expression at the transcription level. In the presence of transcription inhibitor, Actinomycin D, SM22 overexpression was unable to repress the expression of *Nik* after treatment with Actinomycin D for 4 hours (Fig 2D, lane 3&4), suggesting that the regulation of *Nik* by SM22 occurs at transcriptional level. Indeed, we found that SM22 overexpression reduced *Nik* mRNA levels by the qRT-PCR assay (Fig 2E). Taken together, these results suggest that SM22 represses NIK expression at the transcriptional level under basal condition and SM22 inhibits NIK protein degradation under inflammatory condition.

### SRF binds to the *Nik* promoter to increase *Nik* transcription in VSMCs

**Fig 3:**
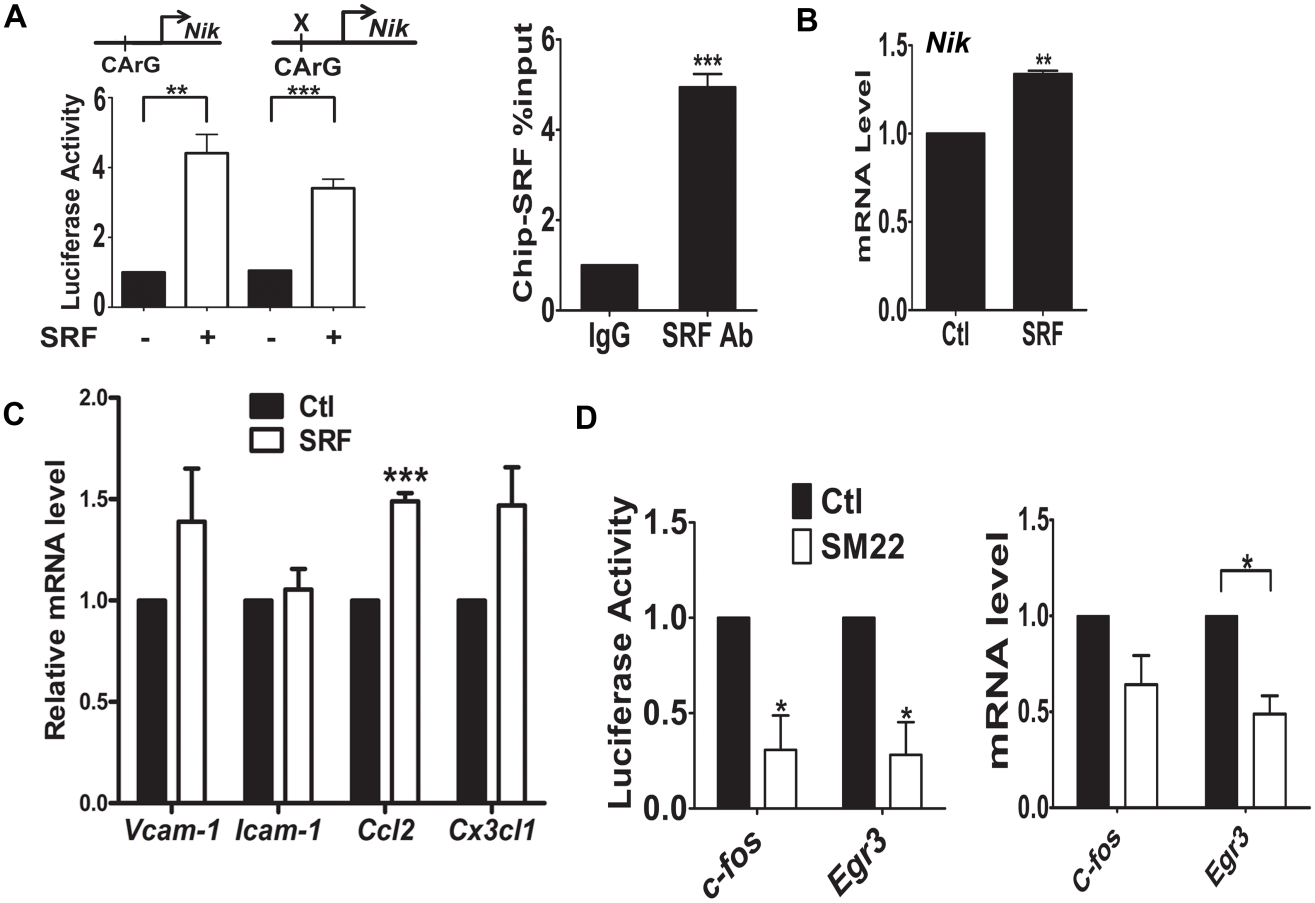
SRF binds to the promoter of *Nik* to stimulate the transcription of *Nik* in VSMCs. PAC1 cells were transfected with indicated plasmids (A) Left panel: SRF transactivated the 2kb *Nik* promoter containing an evolutionarily conserved CArG box at -1.58 kb upstream of the ATG translation initiation site of *Nik* in the rat genome by the luciferase assay. Such transactivation was reduced when the CArG box was mutated. Right panel: SRF bound to the chromatin containing the CArG box in the *Nik* promoter by the ChIP assay. n = 3. (B) Transfected SRF stimulated the transcription of *Nik* by the qPCR assay. n = 4. (C) SRF stimulated the transcription of proinflammatory genes by the qPCR assay in PAC1 cells transfected with the SRF expression plasmid or the empty vector as the control (Ctrl). n = 3. (D) In PAC1 cells transfected with SM22 for 48 hours, SM22 repressed the transactivation of SRF on *c-fos* and *Egr3* promoters and their transcription by luciferase reporter assay and qPCR assays respectively. n = 3. Note: *, **, and *** indicate *p*<0.05, *p*<0.01, and *p*<0.001 respectively vs. the control (Ctrl).

To search for the potential transcription factor that regulates *Nik* transcription, we scanned for potential transcription factor binding sites in the *Nik* promoter using the Promo 3.1 program. We found a conserved CArG box (the SRF binding site) at around 1.58kb upstream of the *Nik* translation initiation site in the *Nik* promoter in the rat genome (S2 Fig). Luciferase assays show that the identified CArG box was responsive to SRF and mutation at the CArG box reduced SRF-induced *Nik* promoter activities (Fig 3A, the left panel). Chromatin Immunoprecipitation (ChIP) assays using the SRF antibody confirmed that SRF bound to the CArG box in the *Nik* promoter in PAC1 cells (Fig 3A, the right panel). Indeed, SRF overexpression upregulated *Nik* transcription (Fig 3B). Also SRF overexpression increased the expression of inflammatory markers in absence and presence of LTβR-Fc (Fig 3C & S3 Fig). These results support *Nik* as a transcriptional target gene of SRF.

To determine whether the inhibitory effect of SM22 on SRF-mediated transcription of *Nik* can be extended to other SRF target genes, we carried out luciferase reporter assays on known SRF target gene promoters such as *c-fos* and *Egr3* [35, 36]. Consistent with our speculation, we found that SM22 overexpression suppressed SRF regulated promoters and their transcription respectively by the luciferase reporter assay (Fig 3D, the left panel) and the qPCR assay (Fig 3D, the right panel).

### SM22 interacts with SRF complex in both the cytoplasm and the nucleus of VSMCs

**Fig 4:**
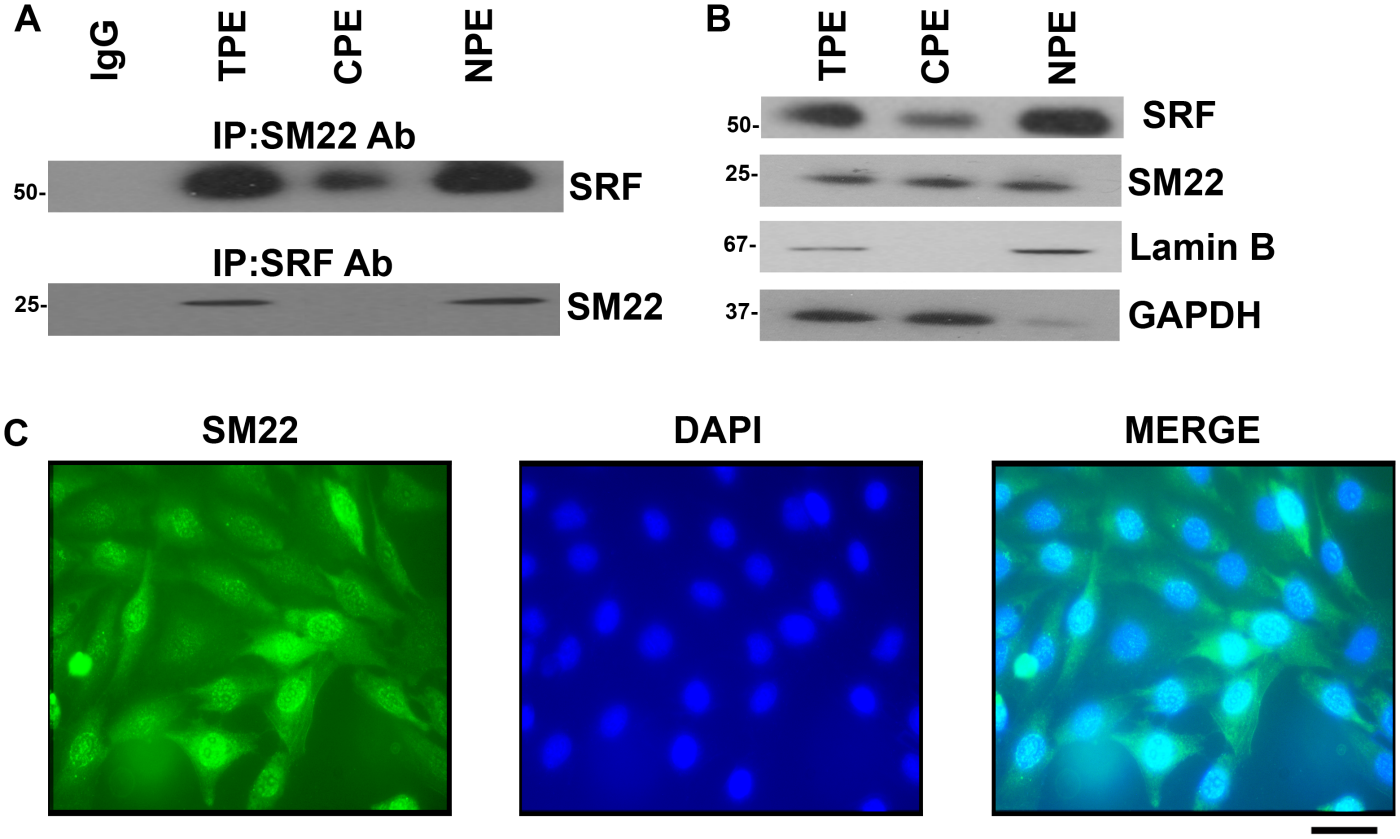
SM22 interacts with SRF and SM22 is expressed in both the cytoplasm and the nucleus in VSMCs. (A) Upper panel: SRF was detected in SM22 immunoprecipitates from both the cytoplasm and the nucleus by co-IP assays using SM22 antibodies followed by WB using SRF antibodies in total protein extract (TPE), cytoplasmic protein extract (CPE) and nuclear protein extract (NPE) from PAC1 cells. Lower panel: conversely, SM22 was also detected in SRF immunoprecipitates from NPE using the SRF antibody. (B) SRF and SM22 are detected in both the cytoplasm and the nucleus of PAC1 cells by WB using the SRF antibodies and SM22 antibodies respectively. Lamin B: the nuclear protein marker; GAPDH: the cytoplasmic protein marker. (C) Immunofluorescence assay using the SM22 antibody detected SM22 expression in both the cytoplasm and the nucleus of PAC1 cells. Scale bar: 20μm. For immunoprecipitation, western blot and immunofluorescence assays: n = 3. The molecular weight markers are indicated on the left side of the WB images. Note: *, **, and *** indicate *p*<0.05, *p*<0.01, and *p*<0.001 respectively vs. the control (Ctrl).

To determine whether SM22 directly affects SRF function, we performed co-immunoprecipitation assays using total cell lysate (TPE), cytoplasmic (CPE) and nuclear protein extracts (NPE) of PAC1 VSMCs. We found that SM22 and SRF formed complexes in both the cytoplasm and the nucleus in PAC1 cells. We detected the presence of SRF in SM22 immunoprecipitates followed by WB using the SRF antibody (Fig 4A). Conversely, we also detected the presence of SM22 in SRF immunoprecipitates in the nucleus, but not in the SRF immunoprecipitates from the cytoplasm (Fig 4A). This might be due to the fact that SRF expression in the cytoplasm is much lower than in the nucleus (Fig 4B).

SM22 (an actin binding protein) is known to be localized in the cytoplasm. However, we observed that SM22 interacted with SRF in the nuclear protein extract. This prompted us to re-evaluate the cellular localization of SM22. WB analyses using fractionated cellular lysates of PAC1 cells revealed that SM22 is localized in the nucleus, not just in the cytoplasm (Fig 4B). The quality of the nuclear protein extract (NPE) and the cytoplasmic protein extract (CPE) were confirmed by the expression of Lamin B (a nuclear protein) and GAPDH (a cytoplasmic protein). Immunofluorescence assays revealed that SM22 was localized in both the nucleus and the cytoplasm of VSMCs (PAC1) using the SM22 antibody (Fig 4C). However, we found that there were heterogeneity of the distribution of SM22 in the nucleus and the cytoplasm among PAC1 cells even in the same culture dish. These results suggest that SM22 interacts with SRF in the nucleus to suppress the transcription of SRF target genes including *Nik*.

### The C-terminal actin binding domain is required for SM22 to suppress inflammation

**Fig 5:**
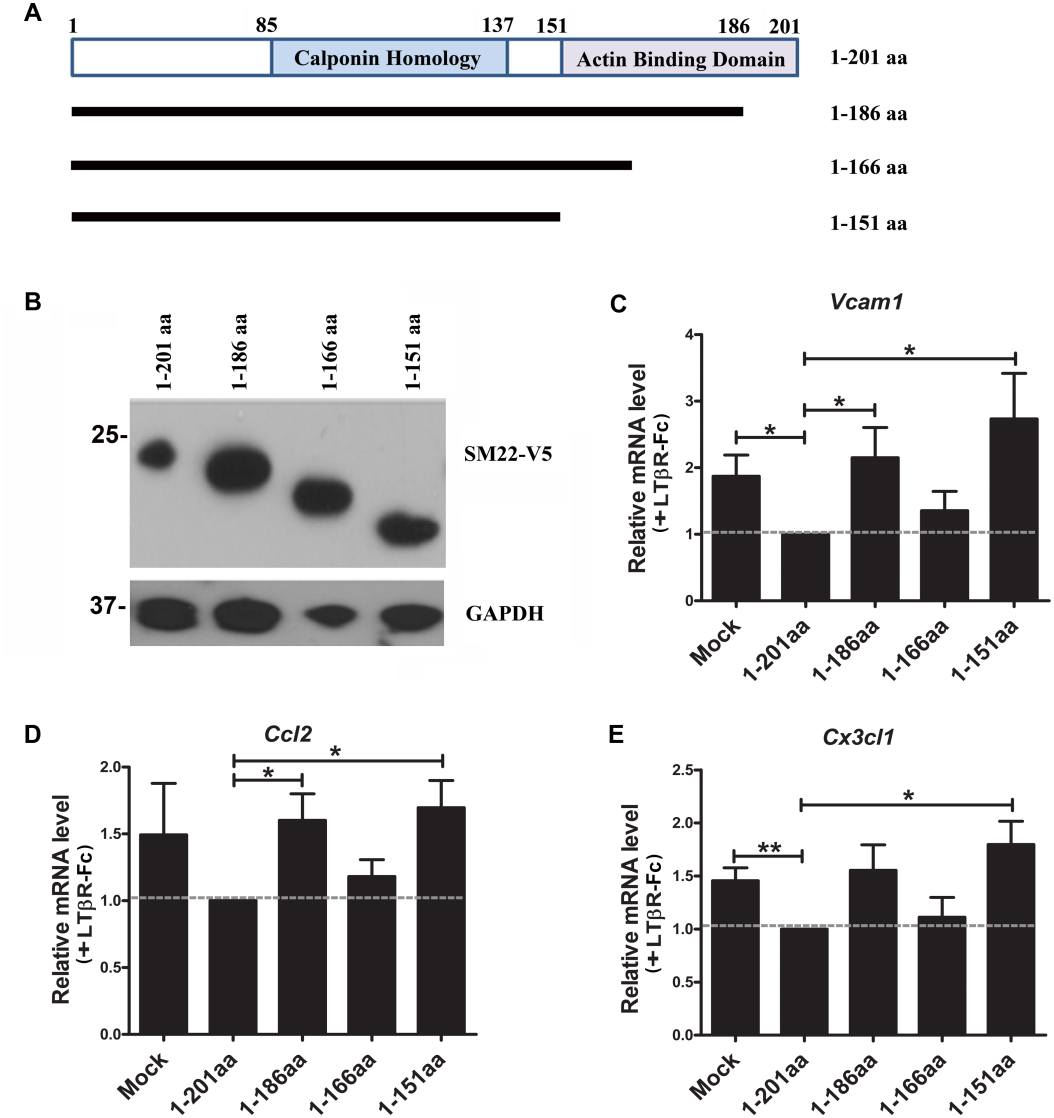
The C-terminal region of SM22 is required for its inhibitory effect on inflammation. (A) Diagram depicting the SM22 protein in full length (1–201) and truncation mutants. (B) The expression of V5-tagged SM22 full length protein (1-201aa) and SM22 truncation mutants was detected in PAC1 cells transfected with the indicated plasmids by WB using the V5-antibody. The molecular weight markers are indicated on the left side of the WB images. n = 4. (C-E) SM22 full length and truncation mutants were transfected into PAC1 cells treated with LTβR-Fc for 24 hours. The effect of SM22 and its mutants on the transcription of inflammatory markers was determined by qPCR assays. The inhibitory effect of the full length SM22 was set to 1 as indicated by the grey dash line. n = 4. Note: *, and ** indicate *p*<0.05 and *p*<0.01 respectively vs. the empty control vector (Ctrl).

SM22 is known to bind to actin via its C-terminal actin binding domain (aa^151-186^) [6]. To determine whether the anti-inflammatory activity of SM22 requires the binding to the actin, we generated a series of C-terminal truncation mutants with V5 tag (Fig 5A). Expressions of these mutants were confirmed by the WB assay (Fig 5B). These mutants were then transfected into PAC1 VSMCs, followed by LTβR-Fc treatment for 24 hours. The expression profiles of inflammatory markers were quantified by qPCR assays. Indeed, the actin binding domain (aa^151-186^) was required for the SM22 anti-inflammatory function (Fig 5C–5E). We do not yet understand why mutant 1-166aa had more inhibitory effect than 1–151 and 1–186 mutants: it is likely that deleting different length of amino acids may have variable effects on their protein structure and thus function.

### SM22 regulates injury-induced NIK expression and inflammation *in vivo*

**Fig 6:**
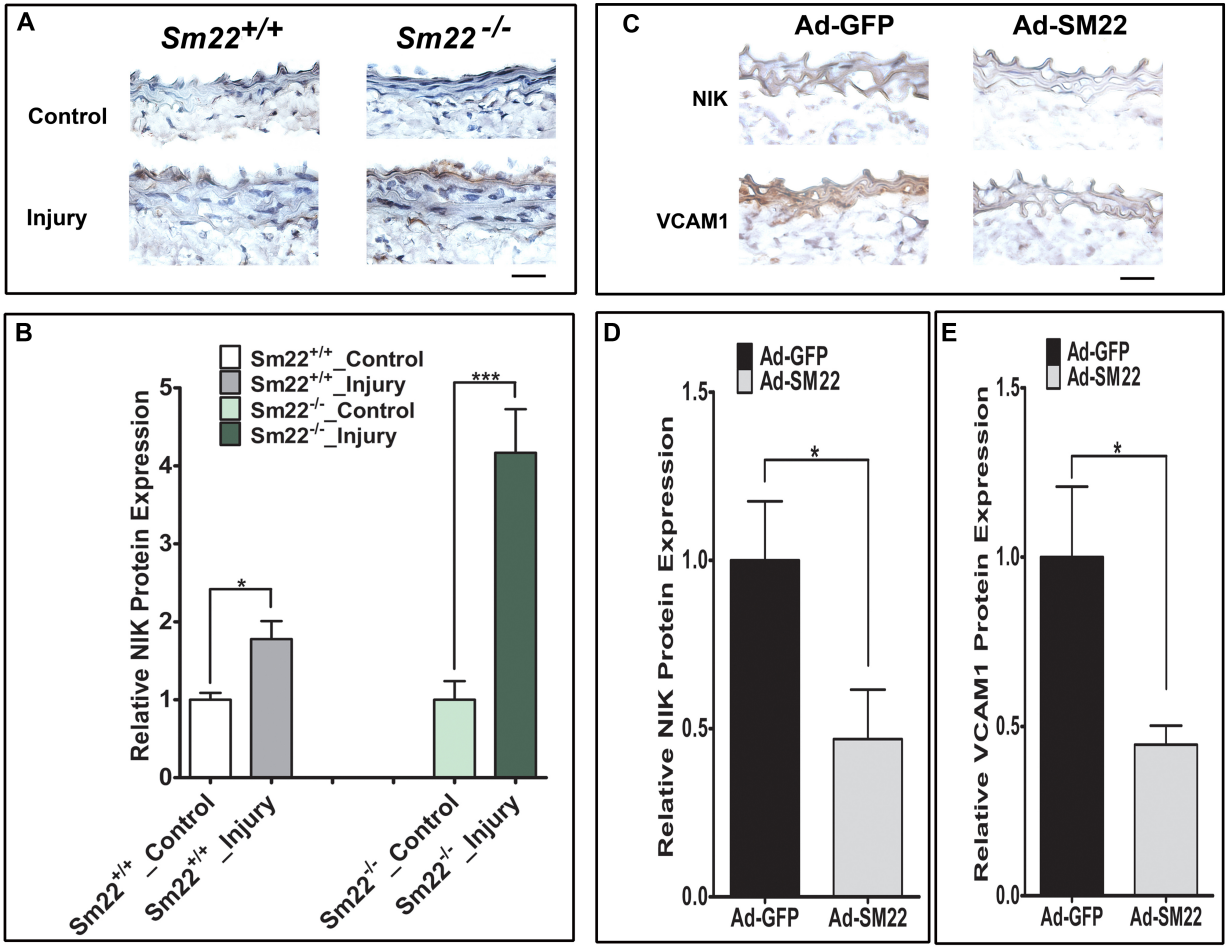
SM22 regulates the expression of injury-induced NIK expression and inflammation *in vivo*. (A) Immunohistochemistry assays show increased NIK expression in carotids of *Sm22*^-/-^ mice compared to wild type *Sm22*^+/+^ control mice two weeks after carotid denudation. The uninjured carotid was used as the control for each mouse. (B) Quantification of NIK signals in the media of the vessel wall using Image Pro software. *Sm22*^+/+^ mice (n = 6); *Sm22*^-/-^ mice (n = 5). (C) The immunohistochemistry assays show decreased expression of NIK and VCAM1 in carotids of *Sm22*^-/-^ mice treated with SM22-GFP adenovirus (Ad-SM22) or its control GFP adenovirus (Ad-GFP) for two weeks after carotid ligation. Scale bar: 20 μm. (D-E) Quantification of NIK or Vcam1 signals in the media of the carotid vessel wall that was transduced with adenovirus Ad-GFP or Ad-SM22. Ad-GFP treated *Sm22*^-/-^ mice (n = 7); Ad-SM22 treated *Sm22*^-/-^ mice (n = 7). Positive signals (the brown stain) at 400X magnification of the carotid images were used for statistical analyses. Values are mean±SEM. *P<0.05, ***p<0.001.

Our previous study shows that SM22 deficiency promotes inflammation and the activation of NF-κB noncanonical signal pathway in the carotids of SM22^-/-^ mice two weeks after carotid injury [12]. To determine whether SM22 regulates the expression of NIK *in vivo*, we performed immunohistochemistry (IHC) assays. We found that SM22 deficiency significantly increased the expression of NIK in injured vessel walls: it is about 1.7 times higher in wild type mice and 4.2 times higher in *Sm22*^-/-^ mice compared with its uninjured control (p<0.5) (Fig 6A and 6B). To confirm that SM22 overexpression inhibits NIK expression and inflammation, we performed carotid ligation in *Sm22*^*-/-*^ mice accompanied by inoculation with adenovirus expressing GFP (Ad-GFP) or SM22-GFP (Ad-SM22). IHC analysis detected the expression of SM22 in injured vessel walls of *Sm22*^*-/-*^ mice infected with Ad-SM22, but not with its control Ad-GFP (S4 Fig). The expression of NIK and VCAM1 was reduced about 53% in the media of the carotid vessel wall in Ad-SM22 infused carotids compared to Ad-GFP controls in *Sm22*^-/-^ mice (Fig 6C–6E). These results demonstrate that SM22 overexpression suppresses NIK expression and inflammation *in vivo*.

## Discussion

**Fig 7:**
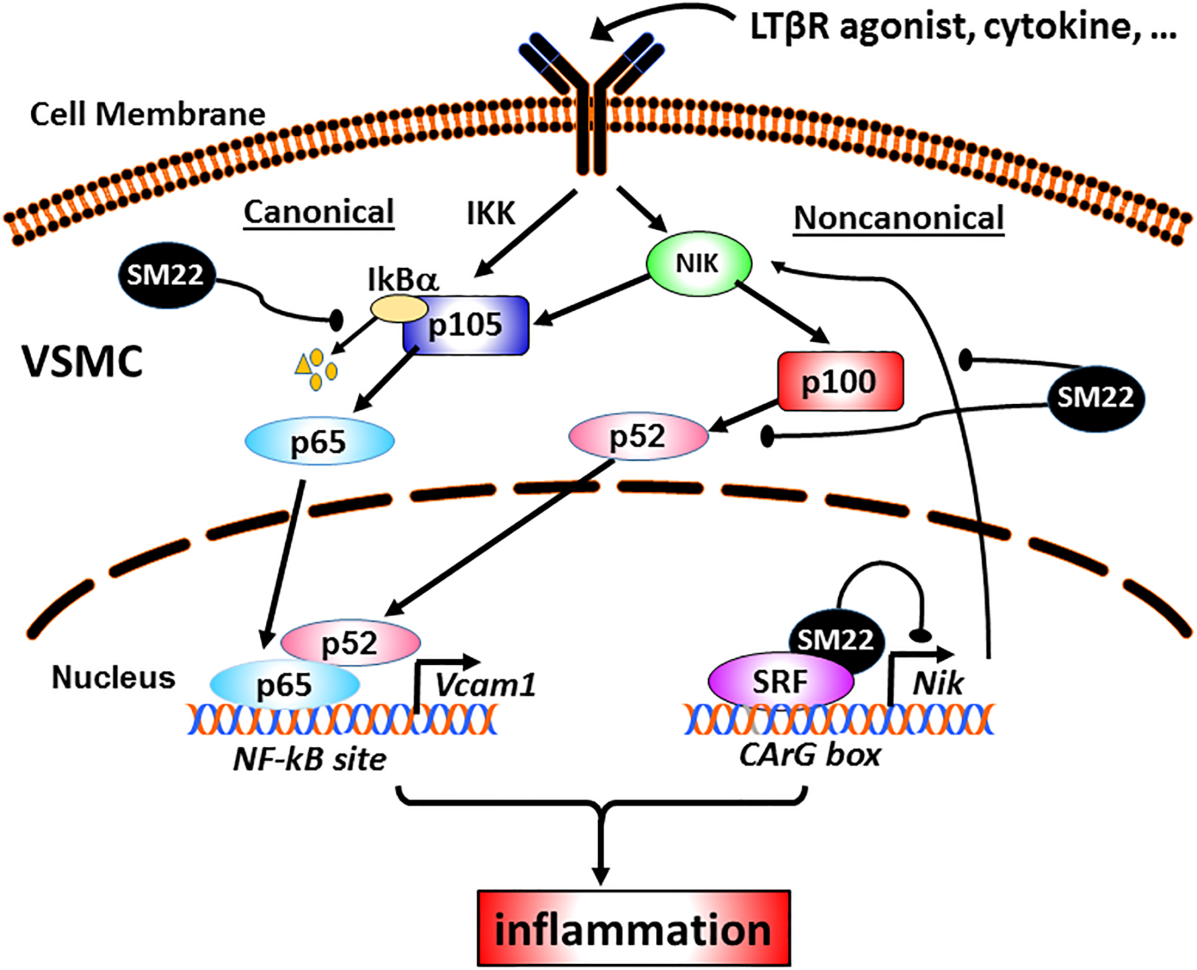
Schematic diagram depicting the regulatory role of SM22 in NF-kB signal pathways in VSMCs under the inflammatory condition. Published studies show that SM22 regulates IKB degradation and p100 proteolysis [12,13]. Here we show that under the inflammatory condition, the activation of proinflammatory cytokine receptor induces both IKK-mediated canonical and NIK-mediated noncanonical NF-κB signal pathways, and thence inflammation. SM22 acts at multiple nodal points along both NF-κB signal pathways from the cytoplasm to the nuclear translocation of NF-κB. In the nucleus, SM22 interacts with SRF to repress the transcription of *Nik* (a SRF binding target) and downstream proinflammatory genes.

SM22, an actin binding cytoplasmic protein, has been widely used as a VSMC differentiation marker [5, 37]. Although Sm22 knockout mice have no apparent abnormality [8–10], our published studies show that after vascular injury these Sm22 knockout mice exhibit increased inflammation through the activation of NF-κB signaling pathways in VSMCs [12]. As a follow-up study to determine the underlying molecular mechanisms, here we report that SM22 acts as an anti-inflammatory protein under inflammatory condition by transcriptionally suppressing *Nik*, an upstream activator of both canonical and non-canonical NF-κB pathways *in vitro* and *in vivo*. A LTβR-Fc, a LTBR agonist, has been used to activate NIK-mediated NF-κB signal pathways [27, 32, 38]. Mechanistically, we discovered an unexpected function of SM22 as a modulator of SRF to repress SRF-mediated *Nik* transcription, leading to reduced inflammation (Fig 7). The present study reveals for the first time that SM22 is expressed in the nucleus in addition to the cytoplasm to regulate the transcriptional activities of SRF in the promoters of SRF targets.

NIK is known to be regulated at the level of protein stability involving proteasome-mediated protein degradation [39]. Using proteasome degradation inhibitor and transcription inhibitor, we found that SM22 regulates NIK expression at both transcriptional level and the protein degradation associated post-translational level in VSMCs depending on the culture condition. It is likely that under the basal condition in the vessel wall, SM22 suppresses *Nik* transcription and thereby NF-κB activation; but upon injury, SM22 downregulation releases the suppression of NIK expression, resulting in the increase of NIK and the activation of NF-κB signal pathways.

We observed that SM22 suppresses inflammation under inflammatory condition, but not under the basal condition: this suggests that SM22 may have a protective role of the vessel wall under pathogenic condition. This is consistent with our published study showing that SM22 knockout mice develop normally but exhibit enhanced inflammation in response to vascular injury [10, 12]. SM22 appears to regulate the activation of NF-κB signal pathways at several nodal points under inflammatory condition. Our previous study show that knockdown of SM22 induces the degradation of IKB (a key regulator for the nuclear translocation of NF-κB) and reduces the proteolysis of p100 [12]. Consistent with this finding, an independent study shows that SM22 inhibits TNFα-induced IκBα degradation [13]. Consistent with these published studies, here we show that SM22 overexpression increases the expression of IκBα and reduces the expression of p105 and p100 under inflammatory condition (Fig 1E). Since the actin binding domain is required for SM22 anti-inflammation function (Fig 5), we will explore the role of actin cytoskeleton in mediating SM22 function in inflammation in future studies.

In elucidating the mechanism of how SM22 transcriptionally regulates *Nik*, we found that SRF binds to the *Nik* promoter to regulate its transcription (Fig 3). SRF is known to be a master regulator for SMC differentiation and dedifferentiation [37]. SRF activity is regulated by a variety of mechanisms involving transcription, post-transcription, nuclear translocation, cofactor availability, posttranslational modifications and protein degradation [2, 35, 40, 41]. We will explore the molecular mechanisms whereby SM22 modulates the function of SRF in VSMCs. Since NF-kB is the master regulator of the transcription of proinflammatory genes, we are interested in investigating the roles of SRF-NF-kB–mediated regulatory network in the transcription of proinflammatory genes.

In this report, we discovered a new mechanism through which SM22 interacts with SRF to regulate the transcription of *Nik*, a key regulator upstream of the canonical and noncanonical NF-κB pathways in a VSMC line. However, this mechanism has not yet been confirmed in animal yet. Further validation of this mechanism in the vessel wall under pathogenic situations will overcome this limitation. The outcome of the *in vivo* validation may have implication regarding seeking optimal methods in the replacement, refinement or reduction (the 3Rs) of the use of animals in research.

Although the finding of nuclear SM22 in SMCs was unexpected, SM22 has been found to be expressed in the nuclei of many cell types such as embryonic ventricle cardiac cells [42] and cancer cells [43]. However, the function of SM22 in the nucleus of these cells has not been determined. Previous studies did not recognize nuclear SM22 in SMCs although reevaluation of previous IF results on SM22 expression in VSMC cells cannot in fact exclude the presence of SM22 in the nucleus [14]. It is likely that the distribution of SM22 in the cytoplasm and the nucleus varies with cell types and their culture conditions. Finally, we would like to point out that increasing evidence shows the nuclear localization of actin binding proteins such as α-actinin, filamin and spectrin [44]. It will be of great interests to determine if other nuclear actin-binding proteins regulate the expression of genes associated with inflammation.

In summary, in this study, we provide evidence supporting the roles of SM22 as an anti-inflammatory protein in VSMCs under inflammatory condition. Importantly, this study revealed a new molecular mechanism whereby SM22 interacts with SRF in the nucleus to modulate the transcription of *Nik*, a key upstream activator of proinflammatory NF-κB signal pathways. Since dysregulation of SRF and NIK has been implicated in many inflammatory diseases including hypertension, restenosis, atherosclerosis, aneurysms and cancer, the role of SM22 in the pathogenesis of these diseases warrants further investigation.

## Supporting information

S1 Fig SM22 overexpression does not suppress inflammation under the basal condition. PAC1 cells were transfected with either SM22 expression plasmid or its empty vector control (Ctrl) plasmid. The effect of SM22 overexpression on the transcription of inflammatory markers was examined by qPCR assays. n = 3. Note: * indicate *p*<0.05 vs. the control (Ctrl).
(PDF)

S2 Fig An evolutionarily conserved CArG box is identified in the *Nik* promoter of human, mouse and rat. In the rat genome, the CArG box is located at -1583bp from the translation initiation site of the *Nik* gene.
(PDF)

S3 Fig Overexpression of SRF increases the expression of proinflammatory marker genes under inflammatory condition. The expression of proinflammatory marker mRNA was determined by the qPCR assay in LTβR-Fc treated PAC1 cells transfected with the plasmid expressing SRF or the empty vector as the control (Ctrl). n = 3. *p<0.05.
(PDF)

S4 Fig The expression of SM22 in the carotids of SM22^-/-^ mice treated with Ad-SM22-GFP adenovirus (Ad-SM22) or its control adenovirus (Ad-GFP). Immunohistochemistry assays using the SM22 antibody show that the expression of SM22 was detected in the vessel wall of the injured carotids from SM22^-/-^ mice infused with Ad-SM22-GFP, but not with its control Ad-GFP. Scale bar: 50 μm. L: lumen of the carotid.
(PDF)

S1 Table Non-standard abbreviations and acronyms. (PDF)

S2 Table Oligonucleotides used in this study. (PDF)
