# Supplementary material for: SM22α suppresses cytokine-induced inflammation and the transcription of NF-κB inducing kinase (Nik) by modulating SRF transcriptional activity in vascular smooth muscle cells
Source: PLoS One. 2017 Dec 28;12(12):e0190191. doi: 10.1371/journal.pone.0190191 (PMC5746259; doi:10.1371/journal.pone.0190191)

S1 Fig. SM22 overexpression does not suppress inflammation in PAC1 cells under the basal condition. PAC1 cells were transfected with either SM22 expression plasmid or its empty vector control (Ctrl) plasmid. The effect of SM22 overexpression on the transcription of inflammatory markers was examined by qPCR assays. n=3. Note: * indicate p<0.05 vs. the control (Ctrl).

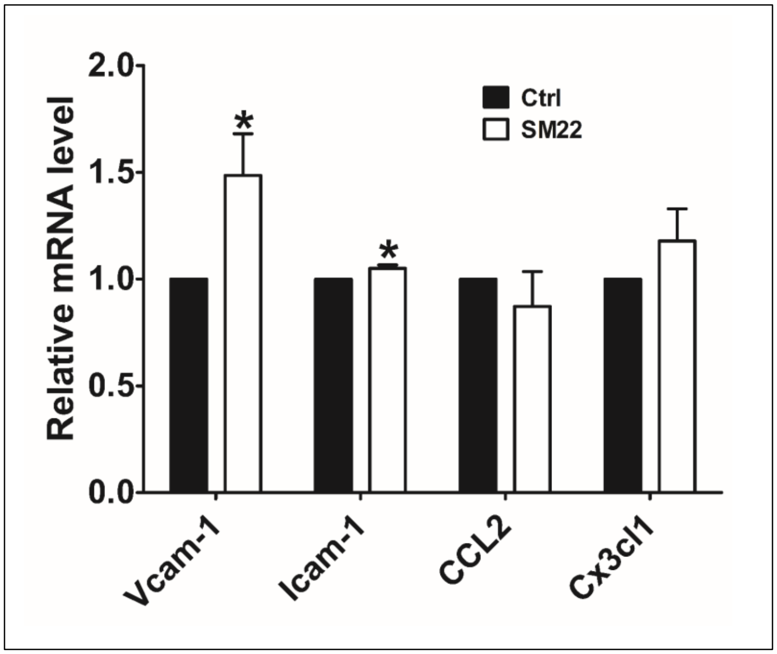

Supplement: S1 Fig — PAC1 cells were transfected with either SM22 expression plasmid or its empty vector control (Ctrl) plasmid. The effect of SM22 overexpression on the transcription of inflammatory markers was examined by qPCR assays. n = 3. Note: * indicate p<0.05 vs. the control (Ctrl). (PDF) [file pone.0190191.s001.pdf]
