# Supplementary material for: SM22α suppresses cytokine-induced inflammation and the transcription of NF-κB inducing kinase (Nik) by modulating SRF transcriptional activity in vascular smooth muscle cells
Source: PLoS One. 2017 Dec 28;12(12):e0190191. doi: 10.1371/journal.pone.0190191 (PMC5746259; doi:10.1371/journal.pone.0190191)
Supplement: S2 Fig — In the rat genome, the CArG box is located at -1583bp from the translation initiation site of the Nik gene. (PDF) [file pone.0190191.s002.pdf]

S2 Fig. An evolutionarily conserved CArG box is identified in the *Nik* promoter of human, mouse and Rat. In the rat genome, the CArG box is located at -1583bp from the translation initiation site of the *Nik* gene.

```
                                                       CArG box
Human -1622 TCTTGAACTCCTGGGCTCAA-GTAATCCTTCTGCCTCAGCCTCCCAAAGTGGTGGGATTA -1563
Mouse -1713 TATTCACATCAAGCCATGCATTTCATGACAGGGTGTC---AACCAACAATGGACTGCATG -1654
Rat   -1625 TATTCATATAAAGCCACGCATTTCATGACAGTGTTTC---CACCAACAATGGACTGCATG -1566
            * ** *  *   *      *  * **      *  **     ** * * ***   *  *
                                  Nik promoter
```
