# Supplementary material for: SM22α suppresses cytokine-induced inflammation and the transcription of NF-κB inducing kinase (Nik) by modulating SRF transcriptional activity in vascular smooth muscle cells
Source: PLoS One. 2017 Dec 28;12(12):e0190191. doi: 10.1371/journal.pone.0190191 (PMC5746259; doi:10.1371/journal.pone.0190191)

S3 Fig. Overexpression of SRF increases the expression of proinflammatory marker genes under inflammatory condition. The expression of proinflammatory marker mRNA was determined by the qPCR assay in LTβR-Fc treated PAC1 cells transfected with the plasmid expressing SRF or the empty vector as the control (Ctrl). n=3, *p<0.05.

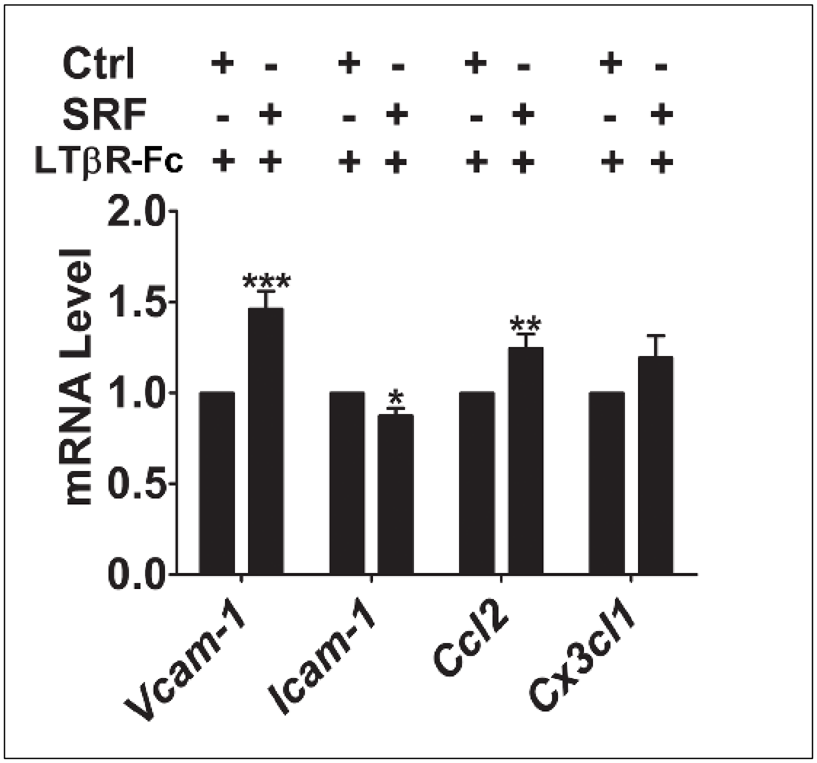

Supplement: S3 Fig — The expression of proinflammatory marker mRNA was determined by the qPCR assay in LTβR-Fc treated PAC1 cells transfected with the plasmid expressing SRF or the empty vector as the control (Ctrl). n = 3. *p<0.05. (PDF) [file pone.0190191.s003.pdf]
