# Supplementary material for: SM22α suppresses cytokine-induced inflammation and the transcription of NF-κB inducing kinase (Nik) by modulating SRF transcriptional activity in vascular smooth muscle cells
Source: PLoS One. 2017 Dec 28;12(12):e0190191. doi: 10.1371/journal.pone.0190191 (PMC5746259; doi:10.1371/journal.pone.0190191)

S4 Fig. The expression of SM22 in the carotids of SM22$^{-/-}$ mice treated with Ad-SM22-GFP adenovirus (Ad-SM22) or its control adenovirus (Ad-GFP). Immunohistochemistry assays using the SM22 antibody show that the expression of SM22 was detected in the vessel wall of the injured carotids from SM22$^{-/-}$ mice infused with Ad-SM22-GFP, but not with its control Ad-GFP. Scale bar: 50 um. L: lumen of the carotid.

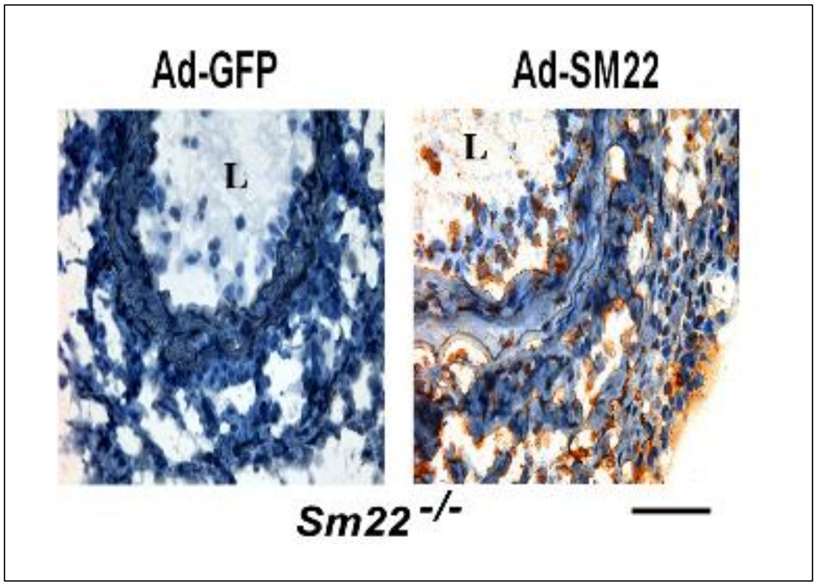

Supplement: S4 Fig — Immunohistochemistry assays using the SM22 antibody show that the expression of SM22 was detected in the vessel wall of the injured carotids from SM22-/- mice infused with Ad-SM22-GFP, but not with its control Ad-GFP. Scale bar: 50 μm. L: lumen of the carotid. (PDF) [file pone.0190191.s004.pdf]
