# Supplementary material for: SM22α suppresses cytokine-induced inflammation and the transcription of NF-κB inducing kinase (Nik) by modulating SRF transcriptional activity in vascular smooth muscle cells
Source: PLoS One. 2017 Dec 28;12(12):e0190191. doi: 10.1371/journal.pone.0190191 (PMC5746259; doi:10.1371/journal.pone.0190191)
Supplement: S1 Table — (PDF) [file pone.0190191.s005.pdf]

S1 Table. Non-standard Abbreviations and Acronyms

SMC	Smooth Muscle Cell
SRF	Serum Response Factor
Tagln	Transgelin
SM22α	SM22, a 22KD smooth muscle protein (α isoform)
LTβR	Lymphotoxin beta receptor
NF-κB	Nuclear factor kappa-light-chain-enhancer of activated B cells
NIK	NF-κB induced kinase, aka MAP3K14
IκB	NF-kB inhibitor
p65/RELA	Transcription factor that is encoded by the *RELA* gene
p52/NFKB2	Nuclear factor NF-kappa-B p100 subunit
VCAM-1	Vascular cell adhesion molecule 1
Co-IP	Co-immunoprecipitation
ChIP	Chromatin Immunoprecipitation
WB	Western Blot
qRT-PCR	Quantitative reverse transcription polymerase chain reaction
