# Supplementary material for: SM22α suppresses cytokine-induced inflammation and the transcription of NF-κB inducing kinase (Nik) by modulating SRF transcriptional activity in vascular smooth muscle cells
Source: PLoS One. 2017 Dec 28;12(12):e0190191. doi: 10.1371/journal.pone.0190191 (PMC5746259; doi:10.1371/journal.pone.0190191)
Supplement: S2 Table — (PDF) [file pone.0190191.s006.pdf]

S2 Table. Oligonucleotides used in this study.

1. **qPCR reactions**

**Gene**	**Forward (5' to 3')**	**Reverse (5' to 3')**	**PCR size (bp)**
Gapdh	TGAATACGGCTACAGCAACAGGGT	TTGTGAGGGAGATGCTCAGTGTTG	151
Vcam1	TGTGAAGGGATTAACGAGGCTGGA	GCACACTTCCACAAGTACAGGAGA	151
Icam1	ACAGCAGACCACTGTGCTTTGAGA	ACTCGCTCTGGGAACGAATACACA	88
Ccl2	CAGTTAATGCCCCACTCAC	GTTTCTGATCTCACTTGGTTCT	217
Cx3cl1	CCTCGGCATGACGAAATGCAACAT	TCTCCTTTGGGTCAGCACAGAAGT	161
Nik	TCCACCTGTCAGGGAGATTC	CTCCTTTCCAAGGGCTTTTC	167
c-Fos	TTCTCTGTTCCGCTCATGACG	CTTCTCAGTTGCTAGCTGCAATCG	105
Egr3	TGACAATCTGTACCCCGAGGA	GCTAGCCGTGGACGTCTGCGTACTG	322

2. **ChIP qPCR assay**

**Name**	**Forward (5' to 3')**	**Reverse (5' to 3')**	**PCR size (bp)**
Nik	TGTTCAGCCCATTTTTAGGC	TTTAGCATTGTGCGAGTGTC	187
